# Not only dominant, not only optic atrophy: expanding the clinical spectrum associated with *OPA1* mutations

**DOI:** 10.1186/s13023-017-0641-1

**Published:** 2017-05-12

**Authors:** Alessia Nasca, Teresa Rizza, Mara Doimo, Andrea Legati, Andrea Ciolfi, Daria Diodato, Cristina Calderan, Gianfranco Carrara, Eleonora Lamantea, Chiara Aiello, Michela Di Nottia, Marcello Niceta, Costanza Lamperti, Anna Ardissone, Stefania Bianchi-Marzoli, Giancarlo Iarossi, Enrico Bertini, Isabella Moroni, Marco Tartaglia, Leonardo Salviati, Rosalba Carrozzo, Daniele Ghezzi

**Affiliations:** 1 Unit of Molecular Neurogenetics, Fondazione IRCCS Istituto Neurologico ‘Carlo Besta’, via Temolo 4, 20126 Milan, Italy; 2 Unit of Muscular and Neurodegenerative Disorders, Laboratory of Molecular Medicine, Bambino Gesù Children’s Hospital, IRCCS, Rome, Italy; 3 Department of Woman and Child Health, Clinical Genetics Unit, University of Padova, Padova, Italy; 4 Istituto di Ricerca Pediatria, IRP, Città della Speranza, Padova, Italy; 5 Division of Genetics and Rare Diseases, Molecular Genetics and Genomics Unit, Bambino Gesù Children’s Hospital, IRCCS, Rome, Italy; 6 Unit of Child Neurology, Fondazione IRCCS Istituto Neurologico ‘Carlo Besta’, 20133 Milan, Italy; 7 Department of Ophthalmology, Neuro-ophthalmology Unit, IRCCS Istituto Auxologico Italiano, Milan, Italy; 8 Department of Ophthalmology, Bambino Gesù IRCSS Children’s Hospital, Rome, Italy

**Keywords:** OPA1, Optic atrophy, Mitochondrial disorder, Encephalopathy, Recessive trait, Targeted resequencing, WES

## Abstract

**Background:**

Heterozygous mutations in *OPA1* are a common cause of autosomal dominant optic atrophy, sometimes associated with extra-ocular manifestations. Few cases harboring compound heterozygous *OPA1* mutations have been described manifesting complex neurodegenerative disorders in addition to optic atrophy.

**Results:**

We report here three patients: one boy showing an early-onset mitochondrial disorder with hypotonia, ataxia and neuropathy that was severely progressive, leading to early death because of multiorgan failure; two unrelated sporadic girls manifesting a spastic ataxic syndrome associated with peripheral neuropathy and, only in one, optic atrophy. Using a targeted resequencing of 132 genes associated with mitochondrial disorders, in two probands we found compound heterozygous mutations in *OPA1*: in the first a 5 nucleotide deletion, causing a frameshift and insertion of a premature stop codon (p.Ser64Asnfs*7), and a missense change (p.Ile437Met), which has recently been reported to have clinical impact; in the second, a novel missense change (p.Val988Phe) co-occurred with the p.Ile437Met substitution. In the third patient a homozygous mutation, c.1180G > A (p.Ala394Thr) in *OPA1* was detected by a trio-based whole exome sequencing approach. One of the patients presented also variants in mitochondrial DNA that may have contributed to the peculiar phenotype.

The deleterious effect of the identified missense changes was experimentally validated in yeast model. OPA1 level was reduced in available patients’ biological samples, and a clearly fragmented mitochondrial network was observed in patients’ fibroblasts.

**Conclusions:**

This report provides evidence that bi-allelic *OPA1* mutations may lead to complex and severe multi-system recessive mitochondrial disorders, where optic atrophy might not represent the main feature.

**Electronic supplementary material:**

The online version of this article (doi:10.1186/s13023-017-0641-1) contains supplementary material, which is available to authorized users.

## Background

Optic Atrophy 1 (OPA1, OMIM*605290) is a dynamin-related protein of the large GTPase superfamily that locates to the inner mitochondrial membrane and is involved in mitochondrial dynamics and mtDNA maintenance.

Pathogenic mutations in the *OPA1* gene have largely been associated with autosomal dominant optic atrophy (ADOA; OMIM#165500), a visual disorder associated with degeneration of retinal ganglion cells. Its prevalence is estimated at 1/50,000. Classical ADOA typically has early onset, before 10 years of age, and manifests with various levels of visual impairment: bilateral visual loss, dyschromatopsia, centrocecal scotomas and temporal optic disc atrophy. Up to 20% of *OPA1*-mutated patients also develop additional more complex neurodegenerative disorder with extra-ocular manifestations, including deafness [1], chronic progressive external ophthalmoplegia, ptosis, ataxia, peripheral neuropathy and mitochondrial myopathy with multiple mtDNA deletions, leading to a syndromic disease sub-group known as ‘ADOA plus’ (OMIM#125250) [2, 3].

To date, over 300 mutations in *OPA1* have been identified and associated with ADOA and ADOA plus. Half of these variants are predicted to result in a truncated protein producing haploinsufficiency and are usually linked to the classical non-syndromic form of ADOA. The ‘ADOA plus’ phenotype is often associated with dominant missense mutations in *OPA1* [1]. In a few cases, the phenotype is due to compound heterozygous *OPA1* mutations preserving transcript expression [3], and suggestive of recessive or semi-dominant patterns of inheritance [3–5]. Recently, compound heterozygous mutations in *OPA1* have been associated with Behr syndrome (OMIM#210000), a disease characterized by the association of early-onset optic atrophy with spinocerebellar degeneration [6, 7]. Finally, the first homozygous *OPA1* mutation has just been reported, associated with fatal infantile mitochondrial encephalomyopathy, hypertrophic cardiomyopathy and optic atrophy [8]. The clinical spectrum of these emerging double-mutant *OPA1*-related disorders remains to be fully characterized.

We report here three patients with biallelic *OPA1* mutations: a boy showing an early-onset and severely progressive mitochondrial disorder and two girls showing a spastic ataxic syndrome associated with sensory motor peripheral neuropathy, resembling Behr syndrome.

## Methods

### Molecular genetics

Total DNA was extracted from muscle, peripheral blood lymphocytes, fibroblasts from patients and relatives using standard methods. Sequencing of the entire mitochondrial DNA (mtDNA) was performed essentially as previously described [9]. mtDNA content was evaluated by real-time PCR-based quantification (ABI7000 Real-Time PCR System) using specific mtDNA probes (amplicons nt 867-928, 12835-12893) and a standard, single-copy autosomal gene (RNaseP) [10].

For Patients1 and 2, NGS library preparation, sequencing, alignment and variant calling were performed as recently described [11]. Filtering was carried out by applying a series of steps: low-quality variants were filtered out (Illumina Qscore threshold of 20); variants with a minor allele frequency (MAF) >1% in the 1000 Genomes Project (http://www.1000genomes.org), Exome Variant Server (http://evs.gs.washington.edu) and Exome Aggregation Consortium (ExAC: http://exac.broadinstitute.org) databases were discharged; finally, we focused on predicted missense, frame-shift, stop-gain or stop-loss, and splice-site variants.

For proband 3, targeted enrichment and massively parallel sequencing were performed on genomic DNA of the affected subject and their parents. Exome capture was carried out using SureSelect Human All Exon V.4 (Agilent). Sequencing data analysis was performed using an in-house implemented pipeline which mainly takes advantage of the Genome Analysis Toolkit (GATK V.3.5) framework [12], as previously reported [13–15]. Quality filtering of variants were as previously reported [16], and high-quality variants were filtered against public (dbSNP146 and ExAC V.0.3) and in-house (approx. 600 population-matched WES) databases to retain private, rare (MAF <0.1%) and clinically associated variants, which were filtered to retain only those located in exons with any effect on the coding sequence, and splice site regions (variants located from −3 to +8 with respect to an exon-intron junction). Functional annotation of variants was performed using SnpEff V.4.2 and dbNSFP V.2.9 [17–19]. WES statistics are reported in Additional file 1. Functional impact of variants was analyzed by Combined Annotation Dependent Depletion (CADD) V.1.3 and dbNSFP Support Vector Machine (SVM) V.2.9 algorithms [18, 19]. Variant prioritization was performed by GeneDistiller [20]. All variants identified by NGS were validated by Sanger sequencing.

RNA was extracted from skin fibroblasts and 1 μg was used as template for reverse transcriptase PCR (RT-PCR) to obtain full-length cDNA. *OPA1* transcript was amplified and PCR products were sequenced in order to confirm genomic variants and unmask potential events of nonsense mediated decay.

### Yeast studies

A hybrid gene was obtained by joining the 5' portion of the yeast *MGM1* gene (the orthologous of human *OPA1*), encoding the mitochondrial targeting sequence and the transmembrane domain, to the 3' of the human *OPA1* cDNA using a sequential PCR protocol. Primers and conditions are available upon request. The resulting construct was then cloned into the pCM184 yeast expression vector. Individual mutants were generated by site-specific mutagenesis.

Yeast strains, media, growth conditions, and the whole procedure for selection of haploid cells have been previously reported in detail [21]. Briefly, a single copy of the *MGM1* gene was inactivated in wild type diploid W303 yeast by homologous recombination with a KANMX4 cassette. Heterozygous strains were transformed with either the wild type or one of the mutant constructs and, after sporulation, haploid yeast harboring the mutant *MGM1* gene and the plasmid of interest were selected and used for subsequent experiments.

### Immunoblot analyses

Fibroblasts were pelleted and solubilized in RIPA buffer with protease inhibitors. 50 μg of proteins were loaded for each sample in 10% denaturing sodium-dodecyl sulfate polyacrylamide gel electrophoresis (SDS-PAGE).

The following antibodies were used: OPA1 (monoclonal antibody BD Biosciences), NDUFA9 (MitoSciences), MTCO2 (MitoSciences), porin/VDAC (MitoSciences), beta-tubulin/TUBB (Sigma-Aldrich), and HSP60 (Abcam).

### Fluorescence microscopy

Skin fibroblasts from patients and controls were cultured in a 37 °C incubator with 5% CO_2_, in either 25 mM glucose or 5 mM galactose DMEM (Euroclone) supplemented with 10% FBS, 1% L-glutamine, and 0.2% sodium pyruvate. For visualization of the mitochondrial network, the mitochondrial fluorescent dye MitoTracker Red-CMXRos (Invitrogen) was added to the culture media at final concentrations of 50 nM for 30 min and then visualized by fluorescence microscopy. Images for P1 were acquired with a confocal microscope (Leica TSC-SP8) and for P3 with inverted microscope (Leica DMi8).

### Biochemical studies

Complex V activity was measured in fibroblast mitochondria of P3 and age-matched controls, using reported spectrophotometric methods [22]. Either succinate (the direct substrate of complex II-succinate dehydrogenase), or malate and pyruvate + malate (which generate NADH, substrate of complex I-NADH dehydrogenase) were used as substrates. Cellular ATP content was assayed luminometrically using the ATPLite 1 Step (PerkinElmer, Boston) according to the procedure recommended by the manufacturer and using 2 × 10^4^ cells. Aged matched controls have been used in three different experiments, either in a regular medium as well as in a galactose-supplemented medium (5 mM). Luminescence was measured using the EnSpire Multimode Plate Readers (PerkinElmer).

### Electrophysiology studies

Electroretinogram (ERG): Retimax instrument (CSO, Firenze, Italy) was used for the full-field flash ERG and pattern VEP assessment in the protocol session. The cornea was anesthetized with 1% dicaine. HKloops ring fiber electrodes were used to record ERGs. A small Ag/AgCl skin earth electrode was placed at the center of the forehead. Mesopic and photopic stimuli were used for recording sessions.

Visual evoked potential (VEP): Visual stimuli consisted of checkerboard patterns (a single check edge subtending 60 and 15 min of arc; contrast 99%; mean luminance 60 cd/m^2^) generated on a monitor subtending 26° and reversed in contrast at the rate of two reversal per second. The stimulation was monocular, with full occlusion of the fellow eye. To maintain stable fixation, a small red target was (0.5°) was placed in the center of the stimulation field. VEPs were recorded by cup-shaped Ag/AgCl electrodes placed over the scalp two cm above the inion (Oz) with the reference in Fpz and the ground on the mastoid. The time-to-peak (in milliseconds) and peak-to-peak amplitude (in microvolts) of major VEP components (i.e. N75, P100 and N145) were measured.

#### Optical coherence tomography (OCT)

Peripapillar retinal nerve fiber layer (RNFL) thickness was measured with a spectral-domain (SD)-OCT (Optos SD-OCT, Glasgow, UK). All scans were done using an internal fixation target in the OCT device. The fast RNFL scan protocol consisted of three consecutive 360° circular scans with a diameter of 3.4 mm centered on the optic disc. Parameters including average RNFL thickness in four quadrants were generated automatically in the analysis report and compared to control values. Each sector was coded in green, yellow, or red for RNFL measurement greater than the lower 95th percentile, less than the lower 95th percentile, or less than the lower 99th percentile range, respectively. All images had signal strength of at least 7. Images with motion artifacts were discarded and rescanned.

## Results

### Case reports

P1. The proband P1 (II-2) is a boy, second child from unrelated parents.

His older brother (II-1) was affected by a neurological disease for which a metabolic etiology was suspected but never confirmed. In II-1, psychomotor delay was reported since first age of life, and he never achieved sitting or standing position. At 9 months of age the presence of ptosis and ophthalmoparesis was depicted, with normal fundus exam. Neurological evaluation at the age 2.5 years showed poor somatic growth (10%ile), microcephaly (<3%ile), hypotonia, ataxia and mental retardation. The following exams resulted normal: plasma lactate, organic acids, amino acids, mucopolysaccharides, oligosaccharides; no brain MRI was available. The sibling II-1 died at age 2 years and 10 months with multiorgan failure during a septic shock due to paralytic ileus. No biological material was available from this individual.

**Fig. 1:**
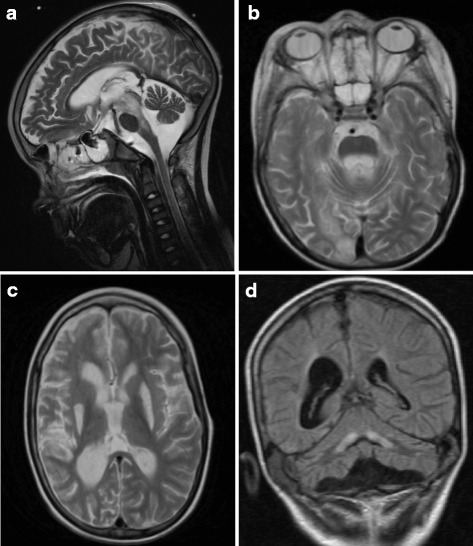
MRI findings of proband 1. **a**, **b**: sagittal and axial T2-weighted sections showing the presence of bilateral hyperintense alterations in the mesencephalon, pons and subthalamic nuclei, all markedly swollen; **c**: axial T2-weighted image showing bilateral necrosis of putamina and left caudate nuclei; **d**: coronal T1-weighted image showing cerebellar atrophy, dentate nuclei hyperintensity and ventricular enlargement

P1 presented since first months with frequent vomiting and marked psychomotor delay: head control was acquired at 10 months, sitting at 2.5 years, standing at 5 years and first words at 3 years of age. He was firstly evaluated at 4 years in another Hospital because of myoclonic epileptic seizures. Clinical examination showed a severe neurological impairment, characterized by absence of head control, poor eye contact and response to sounds, presence of nystagmus, and absence of language. Brain MRI (not available) depicted symmetrical hyperintense alterations in the putamina, cerebellar atrophy and presence of oedema in the occipito-parietal cortical areas. EEG showed poor organization of cerebral activity with the presence of sharp waves in occipital regions. Lactate levels were elevated in plasma (2800 mmol/L) and in CSF; the analysis of respiratory chain complexes and PDH complex on muscle biopsy resulted normal. The neurological conditions were subsequently referred stable, with good control of epilepsy, left hemiparesis and severe mental impairment. Fundoscopic examination at 5 years showed the presence of optic atrophy. At the age of 8 years, following an accidental head trauma, he developed an acute psychomotor regression; 5 months later was admitted to our Institute, showing poor height and growth, scarce response to pain stimuli, absence of postural control, marked hypotonia and absence of spontaneous movements. Hepatic function was markedly impaired; lactate and pyruvate levels were normal. EEG revealed the presence of theta-delta asymmetric activity (depressed on right hemisphere). Brain MRI showed the presence of bilateral, marked and swollen alterations in the mesencephalon, pons and subthalamic nuclei (Fig. 1a, b), associated with necrosis of putamina, and partially in caudate nuclei (Fig. 1c), thin corpus callosum, ventricular enlargement and mild cerebellar atrophy (Fig. 1a, d); the presence of a cortical abnormality in the parietal and occipital right regions was also present. H-MRS showed a very high lactate peak, both in the abnormal and in the spared areas. A second MRI performed after 20 days was unchanged. Neurophysiological study showed the presence of severe axonal sensory neuropathy (absence of Sensory Action Potentials - SAPs, and normal Motor nerve Conduction Velocity - MCV, with mild reduction of Compound Muscle Action Potentials - CMAPs). Respiratory chain complexes and PDH complex activities resulted normal in muscle and fibroblasts. In the following days he developed respiratory failure requiring artificial ventilation and subsequently a severely progressive impairment of clinical conditions with sepsis and a multiorgan failure leading to decease. Parents refused autopsy. Neurological and neuro-ophthalmological evaluations were normal in both parents.

P2. The proband P2 is the only child of unrelated parents of Italian origins. Family history was negative: mother presents severe myopia without optic atrophy or any neurological sign. P2 was born at term after an uncomplicated pregnancy and delivery. Psychomotor development was referred normal. Between ages 4 and 6 years, she presented worsening of her visual acuity. Bilateral optic neuropathy was diagnosed with normal ERG but altered VEPs. She underwent to ophthalmologic follow-up (instrumental data not available). She arrived at our observation at 14 years of age. Neurological evaluation showed mild bilateral ptosis and strabismus, dysdiadochokinesia, dysmetria, mild ataxia and pes cavus; tendon reflexes were absent. Cognitive functions were normal. Fundoscopy and VEPs confirmed optic atrophy; ERG was normal; SAPs and BAEPs showed central conduction abnormalities. Brain MRI disclosed optic nerves and chiasm atrophy. Electroneuronography revealed an axonal sensory neuropathy. Electroencephalography was normal. Plasmatic lactate levels were normal. Treatment with idebenone (135 mg/die) was started. Neurological and ophthalmologic follow-up, six months after diagnosis, were stable.

P3. The proband P3 is a 12 year old girl, who was born at term by dystocic delivery; she was the second child of healthy consanguineous parents. The eldest brother was healthy. She acquired autonomous ambulation at 14 months of age, but parents reported that she had always been unsteady, and had delayed speech development. At 4 years she began to present psychomotor regression, ataxia and deficient motor coordination. At 4 years the neurological examination showed an ataxic-spastic gait, nystagmus, dysmetria and dysarthria. She performed a first brain MRI which showed abnormal hyperintensities of the periventricular and centrum semiovale white matter areas in T2-weighted images bilaterally (Additional file 2). Electrophysiological examinations showed bilateral absence of the V wave at Brainstem Auditory Evoked Potentials (BAEPs), and Nerve Conduction Studies (NCS) disclosed an axonal sensory-motor neuropathy (decreased CMAP and SAP amplitudes with conduction velocities at the lower level of controls range). Phytanic and pristanic acids, VLCFA, Vitamin E, hexosaminidase A and B levels were in the normal range. One year later, the neurological conditions worsened with increased spasticity in her legs and unsteadiness during walking and postural changes. NCS showed a progressive decrease of CMAP amplitudes compared to previous values; VEP and ERG were normal. A second MRI performed at 8 years of age showed T2-hyperintense abnormal signals corresponding to both putaminal nuclei, associated with mild global cerebellar atrophy, while the abnormal areas of T2 hyperintensities of the white matter had disappeared (Additional file 2). Mutations in *FXN, ATM* and *PLA2G6* were ruled out by Sanger sequencing. At age 10 years the neurological examination was stable; repeated flash VEP showed slightly delayed latencies bilaterally, whereas OCT was reported as normal. The last neurological evaluation at age 12 years showed an ataxic-spastic syndrome with distal muscular atrophy of upper and lower limbs. A deep ophthalmological examination was performed: OCT revealed a mild reduction of retinal nerve fiber layer in both eyes whereas flash ERG and pattern VEP responses (60’ stimuli) were in the normal range. However, an amplitude reduction of VEP response was found for 15’ stimuli: abnormalities in VEP 15’ responses, which mainly reflect macular fibers responses contributing to the temporal quadrant of the optic nerve, are quite typical of OPA1- related optic neuropathy (Additional file 3).

### Genetic studies

**Fig. 2:**
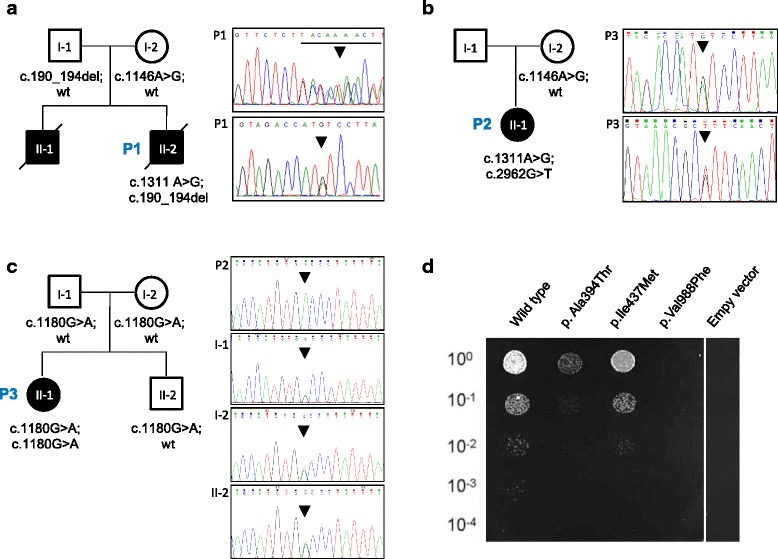
Genetic and yeast studies. **a**, **b**, **c**: Pedigrees and electropherograms showing the mutations found in this study: families of proband P1 (panel **a**), proband P2 (panel **b**) and proband P3 (panel **c**). **d**: Strains lacking *MGM1* and transformed with either wt *OPA1* hybrid allele or individual mutants p.Ala394Thr, p.Val988Phe, p.Ile437Met or empty vector were grown on YP medium supplemented with glycerol. Cells were plated after serial dilutions. Picture was taken after 4 days of growth. Numbering of amino acids in the yeast hybrid genes refers to the corresponding human counterpart (NM_130837.2; NP_570850.2)

Because of the clinical presentation suggesting a mitochondrial disorder, we first investigated the mitochondrial DNA (mtDNA) in our proband P1. The complete sequencing of mtDNA revealed the presence of two homoplasmic variants: the m.﻿11778G > A change in *MTND4*, known to be associated with Leber’s hereditary optic neuropathy (LHON), and the m.﻿﻿3337G > C change in *MTND1.* The latter is expected to cause the p.﻿Val11Leu substitution, affecting a poorly conserved amino acid residue (with leucine present in chicken). Bioinformatics tools for pathogenicity predicted a neutral effect for this amino acid change. Both variants were homoplasmic also in the mother’s DNA. The quality/quantity of the DNA extracted from the proband’s muscle was not enough to perform Southern blot analysis. Since the LHON mutation was unlikely the main cause of the disease in P1, his DNA was subjected to targeted resequencing of a panel containing >100 nuclear genes associated with mitochondrial disorders. After filtering steps to remove common polymorphisms (>1% in public database) and variants with probably neutral effect (synonymous changes, intronic variants >20 bases far from exons), 6 variants remained. Because the pedigree was suggestive for a recessive trait, we focused on the only gene with bi-allelic variants: *OPA1*. Two heterozygous calls were present: a 5 nucleotide deletion c.190_194del (NM_130837.2), predicting a truncated protein product (p.Ser64Asnfs*7), and a previously annotated missense c.1311A > G change (p.Ile437Met). Direct sequencing validated the two variants and confirmed their occurrence in trans, with the missense change on the maternal allele and the frame-shift change on the paternal allele (Fig. 2a). The c.190_194del was absent in public variant databases, whereas the c.1311A > G change is reported with a frequency of 0.06% in ExAc database. The latter corresponds to c.1146A > G, p.I382M based on transcript NM_015560.2, used in the past as reference isoform, and has been already reported in other bi-allelic cases [5, 23].

Targeted sequencing and variant filtering in P2 led to the identification of two heterozygous *OPA1* variants: c.2962G > T (p.Val988Phe), and the recurrent c.1311A > G substitution (p.Ile437Met). Both variants were confirmed by Sanger sequencing; the mother harbored the c.1311A > G, the paternal DNA was not available for genotyping (Fig. 2b).

In P3 data annotation of WES data predicted 13229 high-quality variants having functional impact (*i.e.*, non-synonymous, indels and splice site changes). Among them, 338 private and rare changes were retained for further analyses. Variants were prioritized on the basis of the functional relevance of genes, taking into account both dominant and recessive inheritance models. Only changes predicted to be deleterious by CADD V.1.3 (score >15.0) or dbNSFP SVM V.2.9 (radial score >0.0) algorithms were considered as candidates. Variant filtering and prioritization allowed to identify a missense homozygous variant in *OPA1*, c.1180G > A (NM_130837.2), predicting the p.Ala394Thr amino acid substitution, as the best candidate underlying the trait, which fitted with the documented consanguinity in the family (Fig. 2c). Consistently, no suggestive disease gene candidate with a functionally relevant *de novo* heterozygous variant was identified in the proband (Additional file 1).

### Yeast studies

Yeast lacking *MGM1* cannot grow on non-fermentable carbon sources and develop a petite phenotype [24]. Human *OPA1* per se cannot complement yeast *MGM1* deletion mutants [24]. We therefore employed a hybrid gene comprised of the 5’ portion of *MGM1* (encoding the targeting sequence and the transmembrane domain) and the 3’ portion of human *OPA1* (from exon 6 to the stop codon). Haploid yeast lacking *MGM1* and expressing either the wild-type hybrid gene or individual mutants, were plated at seriate dilutions in medium containing glycerol as sole carbon source (YPD). The wild type hybrid gene restored growth in YPD. Conversely, yeast expressing the p.Ala394Thr displayed a marked reduction of respiratory growth, the p.Val988Phe mutation virtually abolished the activity of the mutant allele, while the defect observed with the p.Ile437Met mutant was milder, evident only at the 10^-3^ dilution (Fig. 2d).

### Characterization of fibroblasts carrying *OPA1* mutations

**Fig. 3:**
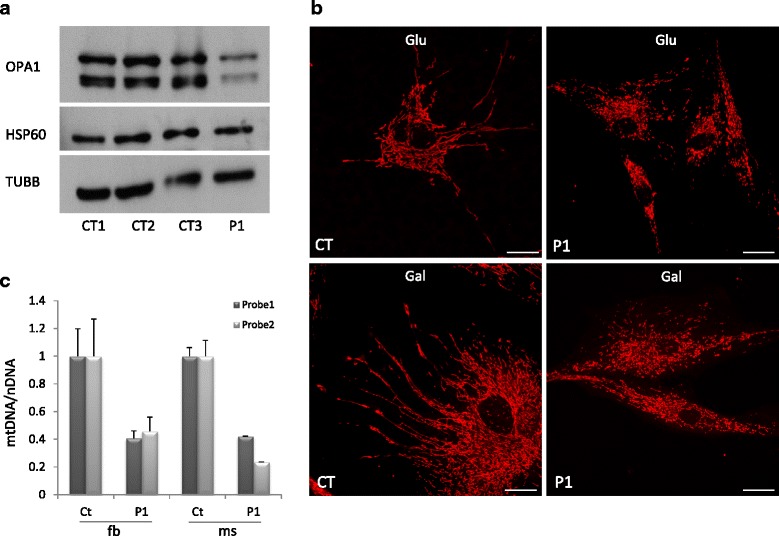
Characterization of fibroblasts from proband 1. **a**: OPA1 protein amount in patient’s (P1) and control (CT1, CT2 and CT3) fibroblasts, obtained using an anti-OPA1 antibody. Anti-TUBB and anti-HSP60 antibodies were used as loading controls. **b**: Representative images of mitochondrial morphology (obtained with Mitotracker *red*) in fibroblasts from proband 1 (P1) and a control (CT), grown either in glucose or galactose medium. Scale bar: 20 μm. **c** Quantification of mtDNA amount in fibroblasts (fb) and skeletal muscle (ms) from proband 1 (P1) and controls. (Ct). The bars represent the amount of mtDNA normalized to nuclear DNA (nDNA), compared to the mean value of controls (=1). Two different probes for mtDNA were used. Data are represented as mean + SD

The *OPA1* transcript levels, assessed by quantitative RTPCR in P1, were comparable in patient’s and control samples and cDNA sequencing indicated that the two alleles were similarly expressed, excluding major mRNA decay of the allele with the premature stop codon. Nevertheless, Western blot analysis on whole cell lysates of primary fibroblasts from P1 showed a clear reduction in the amount of all OPA1 isoforms (Fig. 3a). Given the known role of OPA1 in mitochondrial fusion, to evaluate the functional consequences of the identified *OPA1* variants we assessed the mitochondrial network in fibroblasts. Fibroblasts grown in either standard glucose- or galactose-medium were stained with a mitochondrial dye (Mitotracker Red) and examined by fluorescence microscopy. In patient’s cells we found increased fragmentation of the mitochondrial network compared with controls (Fig. 3b, Additional file 4), in agreement with the expected reduced fusion caused by dysfunctional OPA1. In addition to the role in mitodynamics, OPA1 has been reported to act in the maintenance of mitochondrial genome integrity [25]. In total DNA extracted from fibroblasts we quantified the amount of mtDNA by quantitative PCR. In P1, the mtDNA content was partly but significantly lower than controls (≈40% of the mean control value, Fig. 3c). Notably, the reduction in mtDNA levels (≈35% of the mean control value) was confirmed in DNA extracted from patient’s muscle (Fig. 3c). Similarly, in P3 cells the amount of OPA1 was reduced and the mitochondrial network appears fragmented; as for P1, the latter phenomenon was more evident in medium supplemented with galactose (Additional file 4 and Additional file 5). In the fibroblasts mitochondria of P3 the rate of ATP synthesis was found to be slightly reduced, with respect to the control mean values, when succinate was used, whereas a significant reduction was evident when malate and pyruvate + malate were used as substrates (Additional file 6A). The ATP content in P3 fibroblasts was normal respect to controls in regular medium but significantly reduced in galactose medium reflecting the lower efficiency of ATP production by OXPHOS in stress condition (Additional file 6B). No cells were available from P2.

## Discussion

Compound heterozygous mutations in *OPA1* have been rarely reported, usually associated with ADOA plus phenotype [5, 6, 7, 23, 26]. A table summarizing genetic and clinical features of these cases is reported as Additional file 7. The p.Ile437Met missense variant, although not causing a phenotype *per se*, has been recently suggested to contribute consistently in modulating the phenotype in *OPA1* compound heterozygous subjects [23].

The biallelic *OPA1* variants found in two of our patients combine a classic *OPA1* haploinsufficiency mutation (i.e. a frameshift change in P1 and a missense change, acting as a null allele in yeast, in P2), which can determine dominant isolated optic atrophy with reduced penetrance, and the missense mutation p.Ile437Met, considered hypomorphic or with very low potential pathogenicity since apparently it was not able to lead to clinical symptoms in heterozygous or even homozygous state [23]. Instead, in P3 we found a homozygous p.Ala394Thr change; this is the second case of homozygous missense mutation in *OPA1* reported to date, after the description of a patient with a severe fatal cardiomyopathy associated with the p.Leu589Arg mutation [8].

In accordance with a recessive mode of inheritance, the parents of our probands, each carrier of one heterozygous *OPA1* variant, displayed no symptoms, with the exception of a mother harboring the p.Ile437Met variant and presenting a severe myopia; this genotype-phenotype association was previously reported [5]. Reduced penetrance of dominant mutations, typical of ADOA, may also explain the segregation in the families but it is unlikely given the severe phenotype of these patients.

The effect of the missense mutations on growth of the yeast model seems to correlate with the clinical presentation in biallelic *OPA1* mutant patients; the presence of a virtually null allele (p.Val988Phe found in P2) plus a mildly compromised allele (p.Ile437Met found in P1 and P2) or the presence of a homozygous mutation with an intermediate effect (p.Ala394Thr found in P3) probably cause a similar overall impairment in OPA1 function, resulting in the disease observed in our patients.

From a medical point of view, the most striking aspect of this study is the extremely severe presentation of P1, with an infantile lethal outcome. The same course was reported in the older brother, albeit the genetic diagnosis was not possible because of lack of available DNA. We therefore cannot exclude the presence of a different genetic defect that was the primary cause of the disease in this subject, or even in our proband. However, the extensive genetic analysis with sequencing of >100 genes associated with mitochondrial disorders makes unlikely the latter hypothesis. It is possible that additional genetic factor may have exacerbated the phenotype caused by the *OPA1* mutations. Interestingly, P1 showed two homoplasmic changes in his mtDNA. The mutation m.11778G > A in *MTND4* is a well-known cause of LHON, a mitochondrial disorder that leads to bilateral subacute visual failure. Rarely patients harboring the m.11778G > A show a LHON-plus phenotype; nevertheless visual impairment remains the most common presenting feature, although neurologic features have been documented in several cases, including movement disorders, multiple sclerosis–like illnesses, peripheral neuropathy, and seizures [27]. Haplogroup J has been shown to increase the penetrance of this mutation [28], but our proband belonged to haplogroup T1. However, the low penetrance of the m.11778G > A in females is compatible with the homoplasmic status of the unaffected mother. The other change found in mtDNA, m.3337G > C/p.Val11Leu in *MTND1,* is not phylogenetically conserved, is predicted benign but it is not present in Mitomap database (http://www.mitomap.org). Valine 11 is located at the beginning of the first α-transmembrane helix of ND1 protein, and the change with leucine is not predicted to alter the transmembrane domain. Despite both mtDNA variants affected respiratory complex I, its activity was normal in patient’s samples. All these considerations did not suggest a primary pathogenic role for the two identified mtDNA variants but did not exclude a synergistic effect together with the *OPA1* mutations, worsening the clinical phenotype in our patient.

Amongst the published patients with biallelic *OPA1* mutations (Additional file 7), the severe neonatal-onset disorder characterized by severe optic atrophy, ataxia, hypotonia, gastrointestinal dysmotility and dysphagia, described in two siblings by Schaaf et al. [5], is the closest to the clinical presentation of P1 and his brother. Conversely the phenotype reminiscent of the Behr syndrome, reported in 4 cases by Bonneau et al. [6], was similar to that observed in P2 and P3. However, optic atrophy was evident in all the other *OPA1*-mutant subjects, being often the first and main symptom; in contrast, in our patients overt optic atrophy was observed later compared to most of the other neurological signs (P1 and P3) or not reported (P1’s brother and P3, untill 10 years of age). Moreover, the rapidly progressive course of P1 is reminiscent of Leigh syndrome rather than of DOA-plus or Behr syndrome. Infantile lethal outcome due to *OPA1* mutations has been described for the first time very recently: a homozygous *OPA1* mutation has been found in two sisters with fatal infantile mitochondrial encephalomyopathy, hypertrophic cardiomyopathy and optic atrophy [8]. No evidence of heart involvement was reported in our patients, but a multiorgan failure, including gastrointestinal and hepatic impairment, was observed in P1, confirming that OPA1 proper functioning is crucial not only for the optic nerve but also for several other tissues/organs. Clinical features in P3 were particularly misleading for an *OPA1* related condition, with manifestations of early onset progressive spastic ataxia and sensory motor polyneuropathy, in the absence of optic atrophy. The MRI was likewise peculiar, showing early MRI abnormal hyperintensities of the white matter suggesting a leukodystrophy followed serially by a disappearance of white matter abnormalities and appearance of bilateral abnormalities in the putamen.

## Conclusions

Our report confirms the broad complexity in the phenotypic spectrum associated with recessive *OPA1* mutations that ranges from non-syndromic phenotypes overlapping with those caused by dominant *OPA1* mutations to severe fatal encephalopathy resembling typical mitochondrial diseases. Moreover, our findings suggest that bi-allelic *OPA1* mutations should be considered in disorder where optic atrophy is not obvious (or even absent).

*OPA1* mutations should not be considered only for dominant trait or only for optic atrophy phenotypes.

## Additional files

Additional file 1: Whole exome sequencing data output (table). (DOCX 15 kb)

Additional file 2: Brain MRI of patient 3. (DOCX 1404 kb)

Additional file 3: Ophthalmological examination of patient 3. (DOCX 1304 kb)

Additional file 4: Morphometric analysis of the mitochondrial network in patients’ fibroblasts. (DOCX 114 kb)

Additional file 5: Characterization of fibroblasts from proband 3. (DOCX 884 kb)

Additional file 6: Biochemical characterization of fibroblasts from proband 3. (DOCX 60 kb)

Additional file 7: Genetic and clinical features of patients with biallelic OPA1 mutations (table). (DOCX 15 kb)
