# Supplementary material for: Not only dominant, not only optic atrophy: expanding the clinical spectrum associated with OPA1 mutations
Source: Orphanet J Rare Dis. 2017 May 12;12:89. doi: 10.1186/s13023-017-0641-1 (PMC5427524; doi:10.1186/s13023-017-0641-1)
Supplement: Supplementary file 1 — Whole exome sequencing data output (table). (DOCX 15 kb) [file 13023_2017_641_MOESM1_ESM.docx]

**Additional file 1.** Whole exome sequencing data output.

|  | **P2** |
| --- | --- |
| Target regions coverage, 2x^1^ | 98.8% |
| Target regions coverage, 10x^1^ | 97.8% |
| Target regions coverage, 20x^1^ | 95.5% |
| Average sequencing depth on target^1^ | 95x |
| Number of variants with predicted functional effect | 13,229 |
| Novel, clinically associated, and unknown/low frequency variants^2^ | 338 |
| Putative disease genes (autosomal recessive inheritance)^3^ | 11^4^ |
| Top scored candidate disease genes (autosomal recessive inheritance)^5^ | 4^6^ |
| Genes with putative *de novo* variants^3^ | 2^7^ |

^1^Referred to SureSelect Human All Exon V.4 (Agilent).

^2^MAF <0.1% in dbSNP142 and ExAC V. 0.3 databases, and with frequency <2% in our *in-house* database.

^3^Filtering retained functionally relevant variants by excluding variants predicted as benign by CADD and metaSVM algorithms.

^4^*DNAH7* (c.3139A>G , p.Asn1047Asp; c.5744A>T, p.Asp1915Val), *AHNAK* (c.14896G>A, p.Glu4966Lys), *ATP13A5* (c.2071_2073delCTC, p.Leu691del), *BCL9L* (c.1192G>T, p.Gly398Cys), *CEP164* (c.2326C>T, p.Arg776Trp), *FCER2* (c.559G>A, p.Ala187Thr), *OPA1* (c.1180G>A, p.Ala394Thr), *PERM1* (c.41C>T, p.Pro14Leu), *SAXO1* (c.137C>T, p.Ser46Phe), *WBSCR27* (c.389-3_389-2dupCA), *ZNF569* (c.1042C>T, p.His348Tyr).

^5^Variants were prioritized by means of GeneDistiller using the following keywords: progressive cerebellar ataxia, peripheral axonal neuropathy, speech delay, progressive distal muscular atrophy.

*^6^OPA1* (OS = 17.9), *CEP164* (OS = 8.2), *FCER2* (OS = 2), *AHNAK* (OS = 1).

*^7^AKAP6* (c.4954C>T, p.Arg1652*), *NNT* (c.329dupG, p.Ala111fs). Note that none of the two genes is functionally related to the developmental/cellular processes perturbed in the affected individual.
