# Supplementary material for: Not only dominant, not only optic atrophy: expanding the clinical spectrum associated with OPA1 mutations
Source: Orphanet J Rare Dis. 2017 May 12;12:89. doi: 10.1186/s13023-017-0641-1 (PMC5427524; doi:10.1186/s13023-017-0641-1)
Supplement: Supplementary file 2 — Brain MRI of patient 3. (DOCX 1404 kb) [file 13023_2017_641_MOESM2_ESM.docx]

**Additional file 2**

*
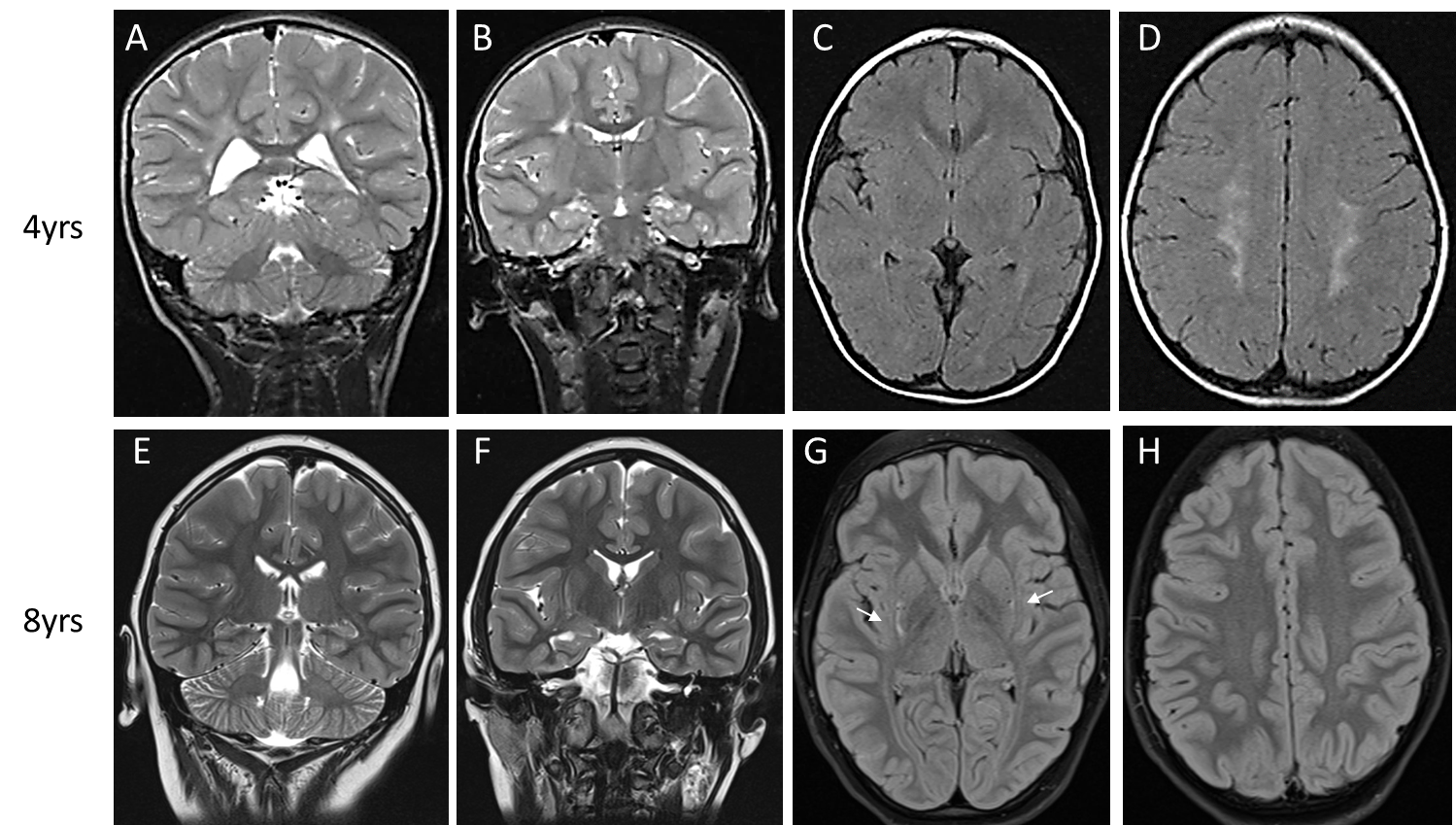
*

**Additional file 2: Brain MRI of patient 2**

A-D. MRI at the age of 4 years; A, B T2 weighted coronal images; C,D FLAIR weighted axial images. Notice periventricular white matter hyperintensities (A,B,D).

E-H. MRI at the age of 8 years; E, F T2 weighted coronal images; G,H FLAIR weighted axial images. White matter hyperintensities have disappeared (E-H); notice moderate cerebellar atrophy (E) and appearance of abnormal small hyperintensities of the pallidum (arrows, G).
