# Supplementary material for: Not only dominant, not only optic atrophy: expanding the clinical spectrum associated with OPA1 mutations
Source: Orphanet J Rare Dis. 2017 May 12;12:89. doi: 10.1186/s13023-017-0641-1 (PMC5427524; doi:10.1186/s13023-017-0641-1)
Supplement: Supplementary file 3 — Ophthalmological examination of patient 3. (DOCX 1304 kb) [file 13023_2017_641_MOESM3_ESM.docx]

**Additional file 3**

**
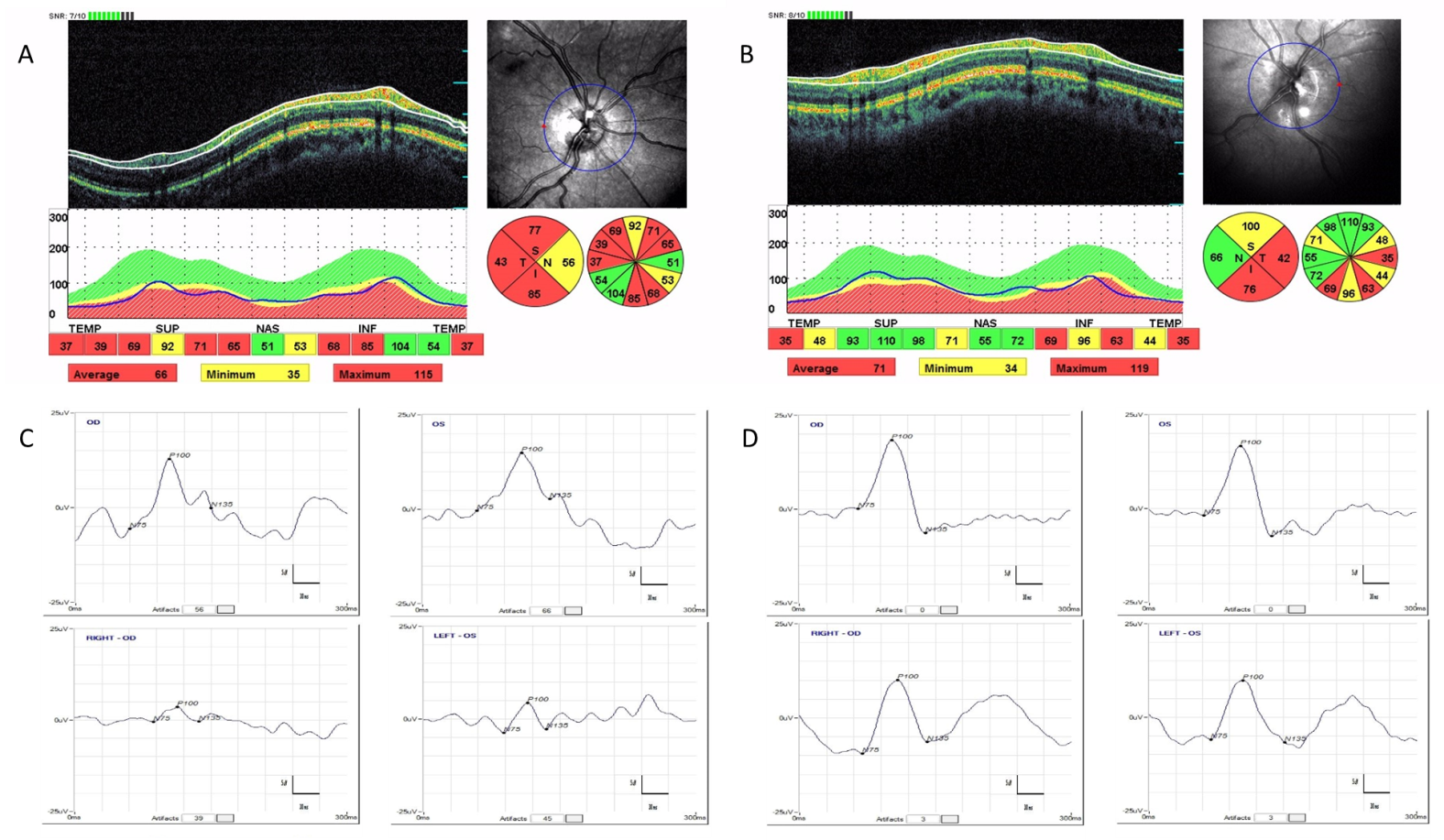
**

**Additional file 3: Ophthalmological examination of patient 3 (performed at 12 years of age)**

A-B. Images of OCT optic nerve scan of right (A) and left (B) eye, showing a mild reduction of Retinal Nerve Fiber Layer in both eyes (RE>LE) mainly in the temporal sector.

C. Pattern VEP responses (60’ stimuli above and 15’ stimuli below) showing normal amplitude and latency response in both eyes for 60’ stimuli. An amplitude reduction was recorded for 15’ stimuli (RE>LE), revealing a mild optic nerve dysfunction in both eyes. Calibration bars: 5 µV for the Y axis and 30 msec of the X axis.

D. Representative examples of normal VEP responses (60’ stimuli above and 15’ stimuli below) of age-matched control. Calibration bars: 5 µV for the Y axis and 30 msec of the X axis.

Visual acuity could not be detected due to the lack of collaboration of the patient.
