# Supplementary material for: Not only dominant, not only optic atrophy: expanding the clinical spectrum associated with OPA1 mutations
Source: Orphanet J Rare Dis. 2017 May 12;12:89. doi: 10.1186/s13023-017-0641-1 (PMC5427524; doi:10.1186/s13023-017-0641-1)
Supplement: Supplementary file 4 — Morphometric analysis of the mitochondrial network in patients’ fibroblasts. (DOCX 114 kb) [file 13023_2017_641_MOESM4_ESM.docx]

**Additional file 4**

A

B


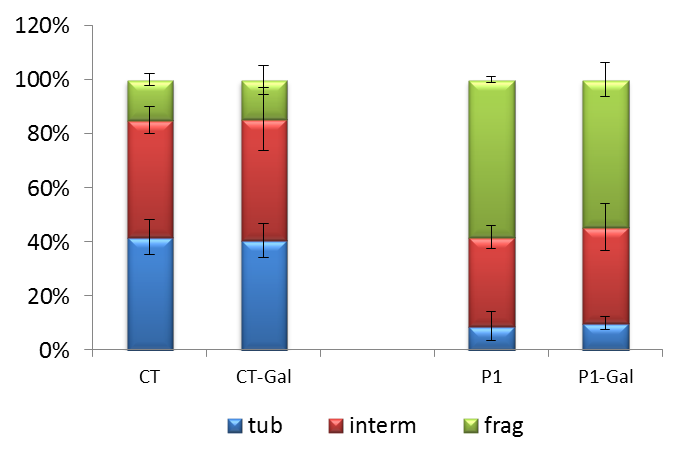

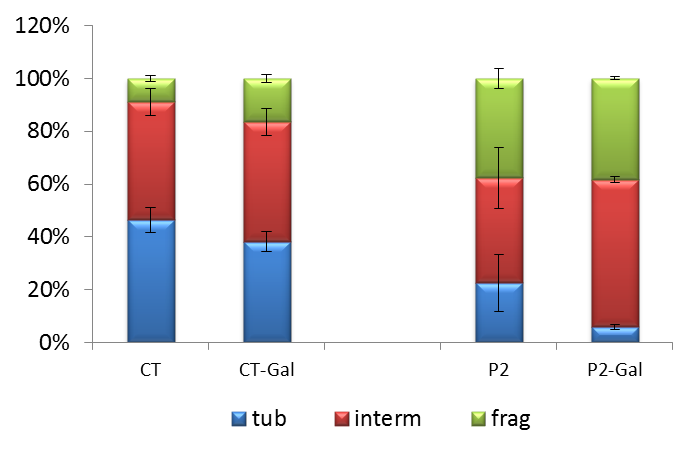


**Additional file 4: Morphometric analysis of the mitochondrial network in patients’ fibroblasts**

Mitochondrial network analysis in fibroblasts from patient 1 (P1, panel A) and 2 (P2, panel B) compared to controls (CT). Fibroblasts grown in either standard glucose- or galactose-medium (Gal) were stained with a mitochondrial dye (Mitotracker Red) and examined by fluorescence microscopy; the mitochondrial network was classified in 3 morphotypes: tubular (tub); intermediate (interm) and fragmented (frag). About 60 cells, from 2 independent experiments, were blindly analyzed for each condition.
