# Supplementary material for: Not only dominant, not only optic atrophy: expanding the clinical spectrum associated with OPA1 mutations
Source: Orphanet J Rare Dis. 2017 May 12;12:89. doi: 10.1186/s13023-017-0641-1 (PMC5427524; doi:10.1186/s13023-017-0641-1)
Supplement: Supplementary file 5 — Characterization of fibroblasts from proband 3. (DOCX 884 kb) [file 13023_2017_641_MOESM5_ESM.docx]

**Additional file 5**

**
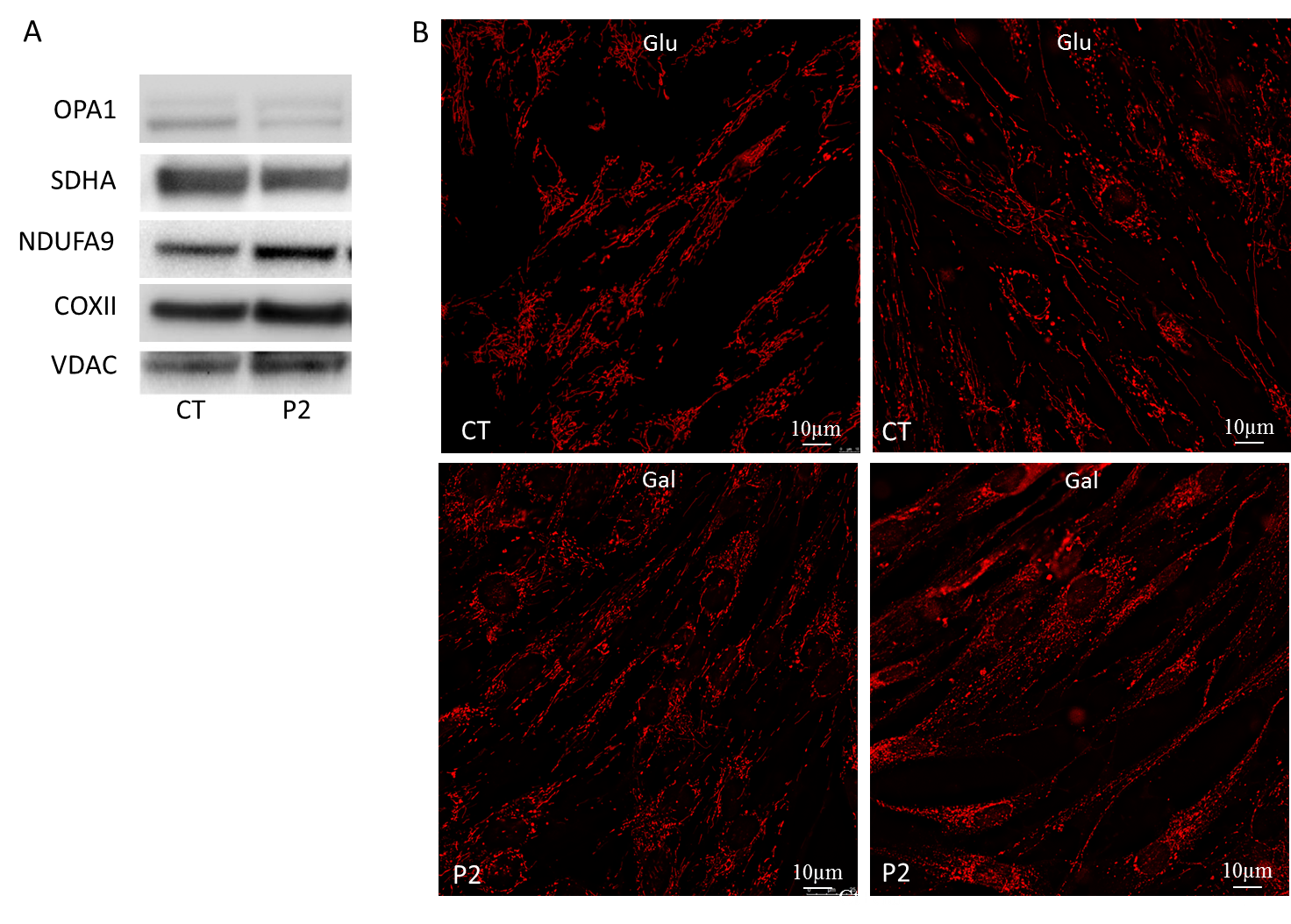
**

**Additional file 5: Characterization of fibroblasts from proband 2**

A: OPA1 protein amount in patient’ (P2) and control (CT1) fibroblasts, obtained using an anti-OPA1 antibody. Anti-VDAC antibody was used as loading control; antibodies against mitochondrial respiratory chain complexes were used (SDHA, for complex II; NDUFAF9, for complex I; COXII, for complex IV).

B: Representative images of mitochondrial morphology (obtained with Mitotracker red) in fibroblasts from proband 2 (P2) and a control (CT), grown either in glucose (Glu, upper panels) or galactose (Gal, lower panels) medium. Scale bar: 10 µm.
