# Supplementary material for: Not only dominant, not only optic atrophy: expanding the clinical spectrum associated with OPA1 mutations
Source: Orphanet J Rare Dis. 2017 May 12;12:89. doi: 10.1186/s13023-017-0641-1 (PMC5427524; doi:10.1186/s13023-017-0641-1)
Supplement: Supplementary file 6 — Biochemical characterization of fibroblasts from proband 3. (DOCX 60 kb) [file 13023_2017_641_MOESM6_ESM.docx]

**Additional file 6**


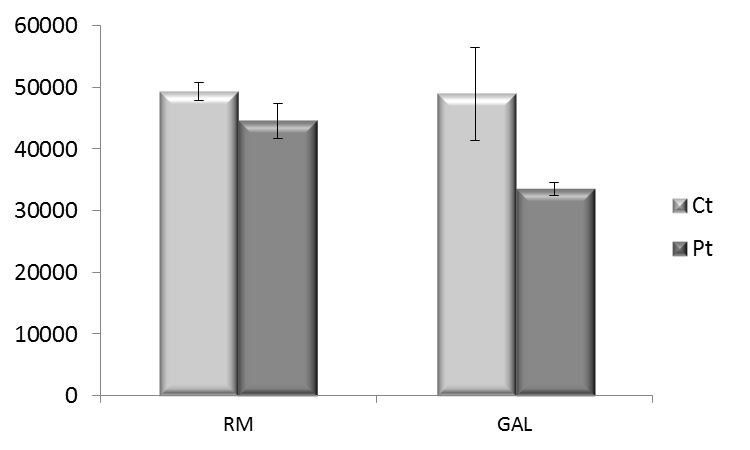


-25%

-32%

a.u. luminescence/mg proteins

nmoles/min/mg protein

CT

P2

-23%

-24%


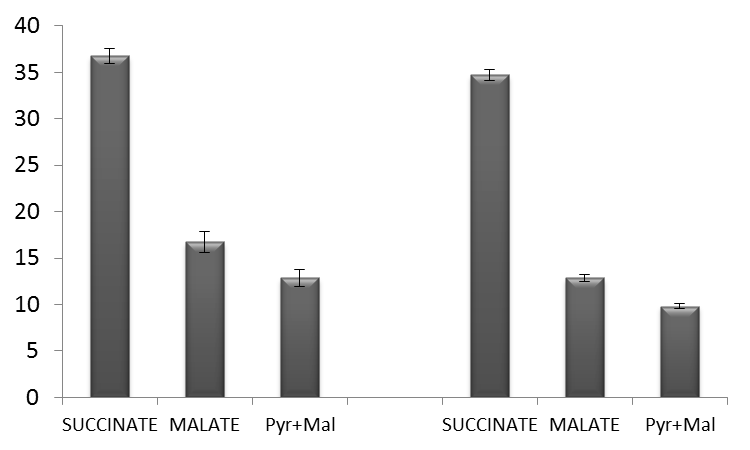


A

B

**Additional file 6: Biochemical characterization of fibroblasts from proband 2**

A: Spectrophotometric determination of complex V activity on mitochondria derived from fibroblasts of patient 2 (P2) and a control (CT). Either succinate, or malate, or malate+pyruvate (pyr+mal) were used as substrate.

B: ATP content assessed by a Luminometric Assay (a.u.: arbitrary units) in control (CT) and patient 2’s (Pt) fibroblasts cultured either in regular medium (RM) or in medium supplemented with galactose (GAL).
