# Supplementary material for: Not only dominant, not only optic atrophy: expanding the clinical spectrum associated with OPA1 mutations
Source: Orphanet J Rare Dis. 2017 May 12;12:89. doi: 10.1186/s13023-017-0641-1 (PMC5427524; doi:10.1186/s13023-017-0641-1)
Supplement: Supplementary file 7 — Genetic and clinical features of patients with biallelic OPA1 mutations (table). (DOCX 15 kb) [file 13023_2017_641_MOESM7_ESM.docx]

**Additional file 7:** Genetic and clinical features of patients with biallelic *OPA1* mutations.

| **Patient**  **[reference]** | **Nucleotide variant** | **Amino acid change** | **Onset** | **Optic atrophy (onset)** | **Other signs** | **Age last examination** |
| --- | --- | --- | --- | --- | --- | --- |
| Proband P1  [present paper] | c.190_194del; **c.1311A>G** | p.Ser64Asnfs*7;  **p.Ile437Met** | < 1 year | Yes (5 yrs) | Hypotonia, ataxia, neuropathy | ┼ 8 years |
| Proband P2  [present paper] | c.2962G>T; **c.1311A>G** | p.Val988Phe; **p.Ile437Met** | 4 years | Yes (6 yrs) | Ataxia, neuropathy | 15 years |
| Proband P3  [present paper] | Homozygous c.1180G>A | Homozygous p.Ala394Thr | 4 years | No | Ataxia, neuropathy | 12 years |
| 2 siblings  [Schaaf et al. 2011] | c.2873_2876del;  **c.1311A>G** | p.Val958Glyfs*3; **p.Ile437Met** | < 1 year | Yes (3 yrs) | Hypotonia, ataxia, dysphagia, gastrointestinal problems | 8 years / 3 years |
| Case 1  [Bonneau et al. 2014] | c.2635C>T;  **c.1311A>G** | p.Arg879*;  **p.Ile437Met** | 1 year | Yes (1.5 yrs) | Ataxia, neuropathy, deafness | 14 years |
| Case 2  [Bonneau et al. 2014] | c.2873_2876del; c.1369G>A | p.Val958Glyfs*3; p.Val457Met | 1 year | Yes (3 yrs) | Ataxia, neuropathy, dysarthria, vomiting | 11 years |
| Case 3  [Bonneau et al. 2014] | c.1834C>T; **c.1311A>G** | p. Arg612* ;  **p.Ile437Met** | 3.5 years | Yes (3.5 yrs) | Ataxia, tremor, neuropathy, constipation | 6 years |
| Case 4  [Bonneau et al. 2014] | c.1624G>A; **c.1311A>G** | p.Glu542Lys; **p.Ile437Met** | 3 years | Yes (3 yrs) | Ataxia, neuropathy | 16 years |
| Family OAK587:  3 cases  [Bonifert et al. 2014] | c.610+364G>A;  **c.1311A>G** | Aberrant transcript; **p.Ile437Met** | 2 years | Yes (2 yrs) | Ataxia, neuropathy, external ophthalmoplegia, muscle atrophy, ptosis | 46 – 48 years |
| Family DUK2976:  1 case  [Bonifert et al. 2014] | c.1316_1317insA;  **c.1311A>G** | p.N440Kfs*14; **p.Ile437Met** | Birth | Yes (birth) | Ataxia, neuropathy | 13 years |
| Single case  [Carelli et al. 2015] | c.1870+1G>T;  **c.1311A>G** | Splicing defect;  **p.Ile437Met** | Birth | Yes (< 1 yr) | Ataxia, neuropathy | 20 years |
| 2 siblings  [Spiegel et al. 2015] | Homozygous c.1766T>G | Homozygous  p. Leu589Arg | Birth | Yes (< 1 yr) | Failure to thrive, hypotonia, neuromuscular weakness, cardiopathy | ┼ 10/11 months |
| Single case  [Lee et al. 2016] | c.2022_2023delinsT;  c.2879G>A | p.Leu675Phefs*13;  p.Arg960Gln | 1 year | Yes (1 yr) | Ataxia, neuropathy, cataract | 10 years |

Nucleotide numbering refers to NM_130837.2; amino acid numbering refers to NP_570850.2. In bold the recurrent *OPA1* variant, frequently found in compound heterozygous subjects. ┼: deceased.
